# Evaluation of Perioperative Beta-Blockers and Factors Associated with Postoperative Atrial Fibrillation in Cardiac Surgery: A Single Center Experience

**DOI:** 10.31083/j.rcm2412370

**Published:** 2023-12-27

**Authors:** Alexandra Puscas, Marius M. Harpa, Klara Brinzaniuc, Hussam Al-Hussein, Hamida Al-Hussein, Cosmin Banceu, Carmen Opris, Claudiu Ghiragosian, Sanziana Flamind, Robert Balan, Septimiu Voidazan, Horatiu Suciu

**Affiliations:** ^1^George Emil Palade University of Medicine, Pharmacy, Science and Technology of Târgu Mureș, 540142 Targu Mures, Romania; ^2^The Department of Cardiovascular Surgery, Emergency Institute for Cardiovascular Diseases and Transplantation Târgu Mureș, 540142 Targu Mures, Romania; ^3^Klinik für Herzchirurgie, Klinikum Passau, 94032 Passau, Germany

**Keywords:** postoperative atrial fibrillation, supraventricular arrhythmia, cardiac surgery, beta-blocker

## Abstract

**Background:**

Postoperative atrial fibrillation (AF) has a complex etiology, and beta-blockers are commonly recommended for its pharmacological prevention. This study aims to assess the impact of beta-blocker therapy on postoperative AF occurrence in patients undergoing aortic valve replacement, mitral valve replacement, surgical revascularization of the myocardium, or a combination of these procedures.

**Methods:**

The study encompassed 472 patients who received aortic valve replacement, mitral valve replacement, surgical revascularization, or their combination. We evaluated the efficacy of preoperative and one-month postoperative beta-blocker administration in preventing postoperative AF, and the associated risk factors involved in the development of postoperative AF.

**Results:**

Of the total patient population, 36% experienced postoperative AF. Our study demonstrated a significant reduction in postoperative AF incidence among patients receiving beta-blocker treatment (all *p*-values < 0.05). Additionally, one-month post-surgery, beta-blocker treatment exerted a protective effect by maintaining the sinus rhythm (*p* = 0.0001). Regarding the risk factors involved in the development of postoperative AF, both age and left atrium (LA) sizeassessed pre-and postoperatively—were positively correlated with the occurrence of postoperative AF (*p* = 0.006). No relationship was found between leukocyte counts and AF incidence. Notably, C-reactive protein (CRP) levels were significantly elevated on the fifth postoperative day in patients with AF (*p* < 0.007). The duration of ischemia was significantly longer in patients with AF (*p* = 0.009).

**Conclusions:**

This study establishes the efficacy of perioperative beta-blocker treatment in mitigating postoperative AF. One month post-surgery, most patients under beta-blocker therapy maintained sinus rhythm, suggesting a potential long-term protective effect of beta-blockers against late-onset AF.

## 1. Introduction

Postoperative atrial fibrillation (POAF) is the most prevalent supraventricular arrhythmia observed after cardiac surgery, affecting roughly one-third of such patients [1]. This condition elevates the risk for stroke and heart failure [2]. Moreover, it is linked with a higher rate of complications, including cardiovascular, cerebral, renal failure, and infections [3, 4]. It’s important to note that while re-intervention for bleeding has been associated with POAF, it may not be the causative agent but rather an exacerbating factor [3, 4]. POAF can be considered the final outcome of multiple predisposing factors, which seem to involve certain pathologies that contribute to the onset of arrhythmia. Furthermore, POAF pathogenesis involves many factors: advanced age, left ventricular dysfunction, dilation of the left atrium, cardiac injury induced by surgical manipulation of the heart, myocardial ischemia, and systemic inflammatory response syndrome caused by cardiopulmonary bypass are may lead to the development of postoperative fibrillation [5, 6]. In addition, patients who develop POAF have significantly higher morbidity and mortality, extended hospitalization days, and higher hospitalization costs [7, 8].

In clinical presentation, POAF often manifests as a tachyarrhythmia that disrupts hemodynamics by shortening the ventricular filling in diastole and coronary flow, thus leading to myocardial ischemia [7, 9]. This is particularly detrimental, especially in patients undergoing surgical myocardial revascularization [7]. Atrial fibrillation (AF) reduces cardiac output by approximately 30%, mainly due to the loss of atrial systole and subsequent suboptimal ventricular filling, factors that can precipitate heart failure [10]. Over time, ventricular remodeling may ensue, significantly elevating the risk of thromboembolic events [9, 10].

The majority of patients undergoing cardiovascular surgery receive chronic drug treatment with beta-blockers, and reintroducing these medications early in the postoperative period has been shown to mitigate adverse events in the sympathetic nervous system, reducing the onset of POAF [11, 12]. The 2020 ESC Guidelines [13] recommend perioperative administration of beta-blockers to patients undergoing cardiac surgery to prevent POAF, as a Class I of Recommendation, Level of Evidence A. Despite these recommendations, the clinical implementation of beta-blocker therapy remains suboptimal, marked by high rates of postoperative discontinuation [14, 15].

In this study, we analyzed the occurrence of POAF and the betablocker prophylaxis in patients undergoing both myocardial revascularization surgery and mitral and/or aortic valve replacement.

## 2. Materials and Methods

**Fig. 1.**
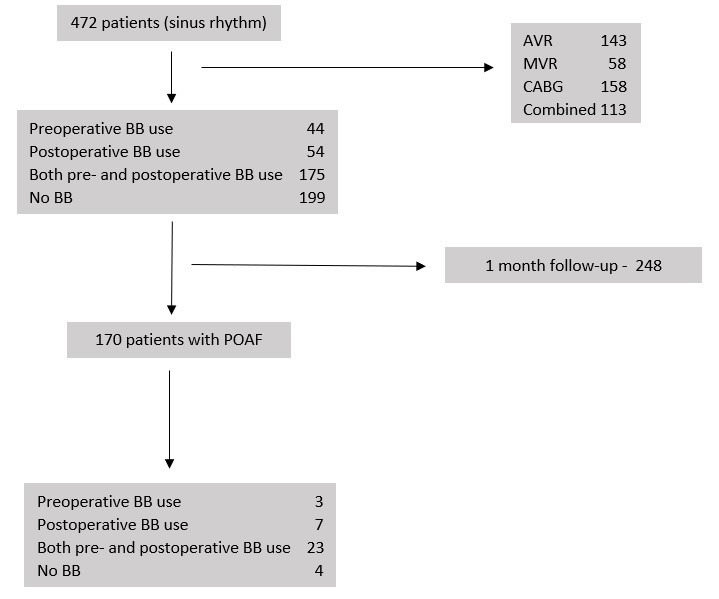
**A flowchart of the patients enrolled in the study**. BB, beta-blockers; AVR, aortic valve replacement; MVR, mitral valve replacement; CABG, coronary artery bypass grafting; POAF, post-operative atrial fibrillation.

A cohort of 472 patients, undergoing mitral valve replacement, aortic valve replacement, surgical revascularization of the myocardium, or a combination of these procedures were prospectively followed at the Emergency Institute for Cardiovascular Diseases and Transplantation Targu Mures, Romania. The study period spanned from October 2020 to May 2023 (Fig. 1). Patients with a history of AF and those who needed a postoperative pacemaker were excluded from the study. Study approval was obtained from the Ethics Committee of the Emergency Institute for Cardiovascular Diseases and Transplantation, Targu Mures, Romania.

The primary objective was to evaluate the efficacy of beta-blockers in preventing POAF. Beta-blockers were used as preoperative (n = 44), postoperative (n = 54) and both pre- and postoperative (n = 175) treatment. We evaluated the incidence of POAF, comparing the patients receiving beta-blockers therapy to a control group that received neither pre- or postoperative beta-blocker therapy (n = 199). Patients were monitored throughout their hospital stay and one month following surgery. Following cardiovascular surgery, oral beta-blocker therapy was initiated during the immediate postoperative period, unless contraindicated due to factors such as hypotension, bradycardia, sinus node disease, grade II/III atrioventricular blocks, or inotropic support). Heart rate was monitored daily through electrocardiography during hospitalization.

The secondary endpoints for assessing POAF risk included cardiac chamber size and ejection fraction (EF), both evaluated using transthoracic echocardiography both pre-and postoperatively. Echocardiographic evaluations were performed one day before surgery and 3–7 days postoperatively. During the examination, the patients were hemodynamically stable, and there was no need for inotropic agents. The levels of potassium, and inflammatory factors (C-reactive protein [CRP] and leukocytes) in the patients were monitored during the postoperative period. The influence of myocardial ischemia time on the occurrence of AF was also evaluated.

All statistical analyses were performed using GraphPad Prism version 8.0.2 (GraphPad Software; San Diego, CA, USA). Statistical significance was set at a *p*-value < 0.05. Chi-square test was used to compare the frequencies of the nominal variables. Quantitative variables were compared according to the data distribution using the *t*-test, and the results were reported as mean ± standard deviation, Mann–Whitney test, and the results were reported as median and range.

## 3. Results

POAF occurred in 36% of patients, with a mean occurrence on postoperative day 2. Patients over 60 years of age were the most affected. There was no statistically significant difference in the gender distribution. The occurrence of AF was not influenced by the type of surgery performed (Table 1).

**Table 1.** **The type of surgery and the occurrence of postoperative atrial fibrillation**.

	AF	No AF	Total	AF (%)	No AF (%)
AVR	49	94	143	34.26	65.73
MVR	24	34	58	41.37	58.62
CABG	51	107	158	32.27	67.72
Combined	46	67	113	40.7	59.29

AF, atrial fibrillation; AVR, aortic valve replacement; MVR, mitral valve replacement; CABG, coronary artery bypass grafting.

Our analysis showed that β-blockers significantly reduce the incidence of POAF when compared to the control group (all *p*-values < 0.05; Table 2). Beta-blockers were typically reintroduced on the second postoperative day. One-month postoperative evaluations were available for 248 of the 472 patients. At this follow-up, only 3.6% (9) of patients exhibited fibrillation, underscoring the protective effect of beta-blocker treatment in maintaining sinus rhythm (*p* = 0.0001).

**Table 2.** **Occurrence of postoperative atrial fibrillation in patients with or without beta-blocker**.

	POAF	No POAF	*p*-value vs. no beta-blocker	OR
Preoperative beta-blocker	7 (15.9%)	37 (84.09%)	0.0006	4.36
Postoperative beta-blocker	15 (27.77%)	39 (72.22%)	0.03	2.14
Preoperative and postoperative beta-blocker	58 (33.14%)	117 (66.85%)	0.02	1.66
No beta-blocker	90 (45.22%)	109 (54.77%)		

POAF, Postoperative atrial fibrillation.

We applied the Student’s *t*-test to independently compare the difference between the means, and we analyzed the association between the dimensions of the left atrium (LA), left ventricle (LV), right ventricle (RV), EF, and cardiac chambers pre-and postoperatively. The size of the LA pre-and postoperative was positively associated with the occurrence of AF. In contrast, pre-and postoperative LV, and RV size did not correlate with the occurrence of AF. The same results were observed for pre-and postoperative EF, without statistical significance (Table 3).

**Table 3.** **Correlations between atrial fibrillation and cardiac chamber size, and EF evaluated pre and postoperatively**.

Preoperative		POAF	No POAF	*p*-value
	LA (mm)	45.15 ± 6.96	43.31 ± 6.9	0.006
	LV (mm)	52.45 ± 7.49	51.61 ± 6.68	0.21
	RV (mm)	33.55 ± 5.89	32.98 ± 5.31	0.28
	LVEF (%)	51.38 ± 8	51.85 ± 8	0.54
Postoperative				
	LA (mm)	44.5 ± 6.58	42.61 ± 6.83	0.007
	LV (mm)	49.92 ± 7	49 ± 6.53	0.17
	RV (mm)	31.87 ± 4.84	31.82 ± 4.76	0.9
	LVEF (%)	49 ± 5.99	49.43 ± 6.55	0.5

All data were expressed as mean and standard deviation. LA, left atrium; LV, left ventricle; RV, right ventricle; LVEF, left ventricle ejection fraction; POAF, Postoperative atrial fibrillation; EF, ejection fraction.

Our analysis showed no significant differences in serum potassium concentrations between patients with and without POAF (all *p* > 0.05; Table 4). The Mann–Whitney test was performed to search for any potential influence of leukocyte levels on the incidence of AF, however there was no correlation between this inflammatory marker and the occurrence of AF. Interestingly, CRP values were significantly higher on the fifth postoperative day in patients with AF (Table 5).

**Table 4.** **Correlations between serum potassium levels and atrial fibrillation**.

Serum potassium concentration	Postoperative day	Postoperative atrial fibrillation		
		Yes	No	*p*-value
	Day 0	3.79 ± 0.47	3.79 ± 0.49	0.91
	Day 1	4 ± 0.34	4 ± 0.37	0.21
	Day 2	3.88 ± 0.38	3.88 ± 0.35	0.97
	Day 3	3.81 ± 0.35	3.85 ± 0.38	0.24
	Day 4	3.79 ± 0.44	3.81 ± 0.4	0.68
	Day 5	3.82 ± 0.43	3.91 ± 0.45	0.09

All data were expressed as mean and standard deviation.

**Table 5.** **Correlations between CRP level, leukocytes and the occurrence of postoperative atrial fibrillation**.

CRP level	Postoperative day	Postoperative atrial fibrillation		*p*-value
		Yes	No	
	Day 1	5.1 (0.64–26.95)	5.58 (0.38–38.81)	0.12
	Day 2	13.58 (2.8–30.91)	15.48 (0–31.84)	0.25
	Day 3	15.15 (1.56–30.98)	15.71 (1.27–38.67)	0.34
	Day 4	12 (1–31.97)	10.32 (0.36–45)	0.31
	Day 5	8.23 (0.89–28)	6.55 (0.36–29.7)	0.007
White blood cell count	Postoperative day	Postoperative atrial fibrillation		*p*-value
		Yes	No	
	Day 0	10.85 ± 3.82	10.72 ± 3.71	0.83
	Day 1	11.1 ± 3.27	11 ± 3.39	0.91
	Day 2	12.58 ± 3.93	12.12 ± 3.85	0.22
	Day 3	11.35 ± 3.86	10.77 ± 3.91	0.19
	Day 4	9.81 ± 3.22	9.4 ± 3.65	0.33
	Day 5	9.42 ± 3.29	9.54 ± 3.86	0.79

All data were expressed as median and interquartile range. CRP, C-reactive protein.

Our data revealed that the mean ischemia time was notably longer in patients with AF compared to those without, with mean durations of 85.89 ± 35.22 minutes and 77.67 ± 31 minutes, respectively (*p* = 0.009). Additionally, hospitalization durations were significantly extended in patients who developed AF, with a median stay of 10 days (ranging from 5 to 34 days), compared to a median of 8 days (ranging from 5 to 67 days) for those without AF (*p* = 0.0001).

## 4. Discussion

The underlying mechanisms of AF, including both intra- and postoperative phenomena, are not completely understood. These mechanisms, which include inflammation, sympathetic nervous system activation, and myocardial ischemia, often interact with pre-existing risk factors like advanced age and atrial dilation to induce AF. Therefore, strategies to prevent AF are only partially effective, with no significant reduction in the incidence of arrhythmias [16, 17].

Adrenergic overstimulation or activation of the sympathetic nervous system can act as a catalyst for AF [18]. The 2020 EACTS Guidelines recommend initiating beta-blocker treatment as soon as possible to mitigate the risk of AF [13]. While ceasing beta-blocker treatment in the immediate aftermath of surgery increases the risk of AF development, timely reintroduction or even new treatment initiation is associated with a lower incidence of such arrhythmic complications [19, 20, 21, 22]. Despite this, the protective benefits of perioperative beta-blocker use against AF appear inconsistent. Although the administration of beta-blocker has increased over time, it hasn’t led to a corresponding decline in the occurrence of AF [23]. A possible explanation could be a rebound phenomenon caused by discontinuation of beta-blocker treatment, which can increase the risk of AF due to the hyperadrenergic status [24, 25, 26]. To avoid this rebound effect, patients on preoperative beta-blocker therapy should receive beta-blockers on the day of surgery and should continue the treatment on the first postoperative day, unless contraindicated [27].

Some studies that evaluated drug strategies, including the administration of beta-blockers as prophylaxis for AF, have shown mixed results regarding their protective efficacy [28]. A study by Kim *et al*. [29] observed administering beta-blockers prior to aortic valve replacement surgery (on the occurrence of AF), did not yield protective effects. However, when beta-blocker therapy was initiated immediately after surgery, a protective effect was observed. Trials avoiding beta-blocker withdrawal still found the treatment to be effective, but less so compared to studies where the treatment was discontinued [30, 31]. Our study corroborates these findings: initiating or continuing beta-blocker treatment reduced both the occurrence of AF and the length of the hospital stay. These findings are consistent with the data from randomized and non-randomized trials from the literature, that are showing lower rates of POAF in those receiving perioperative beta-blocker therapy [32, 33].

At the 1-month follow-up visit, most patients on beta-blocker therapy were in sinus rhythm, and this could suggest a long-term protective effect in maintaining sinus rhythm.

Hypokalemia, which increases myocardial automatism and excitability, can lead to supraventricular and ventricular arrhythmias; therefore, it is considered a risk factor that contributes to AF occurrence [34, 35]. Some studies report that maintaining a serum potassium level of above 4.5 mmol/L reduces the occurrence of AF [36]. Although the involvement of hypokalemia in the pathology of POAF has been recognized, the relationship between potassium levels and AF after cardiac surgery has not been well defined.

Some studies investigating potassium replacement protocols did not detect a decreased risk of atrial tachyarrhythmias [37]. For example, while Lancaster *et al*. [38] found no protective benefits of postoperative potassium supplementation against AF, patients with AF were found to have higher potassium levels. Our study found no link between serum potassium levels and AF occurrence. Patients with and without AF had approximately the same levels of potassium 1–5 days postoperatively. Old age remains a strong risk factor for AF. The process of atrial remodeling, with fibrosis, results in slower atrial conduction, thus increasing the risk of AF [39, 40]. The results of this study are in accordance with those of previous studies.

Atrial dilation, which is considered to be a marker of increased filling pressure, is associated with inflammatory and degenerative changes that cause alterations in atrial electrophysiological properties [41]. Thus constituting a substrate in the development of AF, as confirmed in our study [41]. Both advanced age and left atrial dilation are related to the onset of AF and can be used to stratify the risk of AF [42, 43]. Inflammatory changes induced by the use of cardiopulmonary bypass and myocardial ischemia have also been recognized as factors involved in the pathogenesis of AF, as they are capable of triggering an inflammatory cascade that can induce fibrotic changes in the myocardium, which could stimulate arrhythmogenesis [44, 45]. Some studies suggest that a pronounced increase in leukocyte and CRP levels due to the inflammatory response caused by cardiopulmonary bypass and myocardial ischemia could be considered as predictive factors for the occurrence of AF [46, 47].

In this study, we found no significant correlations between leukocytosis and the incidence of AF, potentially due to the absence of inflammatory syndrome features among the patients. In contrast, CRP levels were significantly higher in patients with AF on the fifth postoperative day. Generally, C-reactive protein reaches a maximum value approximately 2–3 days postoperatively, corresponding to the most frequent onset of AF [48]. While our study demonstrated maximum CRP values on postoperative days 2–3 (in both groups), there was no statistically significant difference between the groups, as both showed similar values. By the fifth postoperative day, patients with AF had significantly higher CRP levels compared to patients without AF, in whom CRP values had progressively decreased. Additionally, our study identified a significant link between myocardial ischemia and AF; specifically patients with AF experienced longer durations of ischemia.

There are several limitations to be noticed in this study. First, the reintroduction of beta-blockers could be better optimized in terms of dosage, titration, drug type, timing to minimize patient variability. Second, further research involving a larger patient cohort and extended follow-up periods are needed to solidify preventative drug strategies. Lastly, additional studies are required to determine whether potassium replacement serves as a protective factor against postoperative POAF and to better understand the role of the systemic inflammatory response syndrome in the development of this condition.

## 5. Conclusions

Current prevention strategies are only partially effective against POAF. Our study demonstrate the efficacy of preoperative or postoperative beta-blocker treatment in the prevention of early POAF. Regarding late-onset AF, most patients at 1 month postoperative mark were maintaining sinus rhythm while on beta-blocker therapy, suggesting long-term protection. While the systemic inflammatory response syndrome is acknowledged as a contributor to the onset or maintenance of AF, the connection between them is not well defined. This study only partially elucidated the involvement of these risk factors to AF development.

In conclusion, given the complex interplay of factors contributing to the onset and maintenance of POAF, beta-blocker-based prevention strategies are only partially effective. This is further compounded by suboptimal adherence to beta-blocker therapy and high rate of postoperative discontinuation.

## Data Availability

All the data can be found in the archive of Emergency Institute for Cardiovascular Diseases and Transplantation Târgu Mureș, Romania.
